# Potential Role of Circulating Biomarkers in Peripheral Artery Disease

**DOI:** 10.1007/s11886-026-02402-3

**Published:** 2026-08-21

**Authors:** Alek C. Keegan, Philip Greenland, Donald M. Lloyd-Jones, Neela D. Thangada

**Affiliations:** 1 Department of Medicine, Feinberg School of Medicine, Northwestern University, Chicago, IL USA; 2 Division of Cardiology, Department of Medicine, Feinberg School of Medicine, Northwestern University, 675 N St. Clair St. Ste 19-100, Chicago, IL 60611 USA; 3 Department of Preventive Medicine, Feinberg School of Medicine, Northwestern University, Chicago, IL USA; 4 Framingham Center for Population and Prevention Science, Section of Preventive Medicine & Epidemiology, Department of Medicine, Boston University Chobanian & Avedisian School of Medicine, Boston, MA USA

**Keywords:** Inflammation, Troponin, N-terminal pro-brain natriuretic peptide, Lipoprotein A, Omics, Major adverse limb events

## Abstract

**Purpose of Review:**

To review evidence on the role of circulating biomarkers associated with lower extremity peripheral artery disease (PAD) and adverse outcomes in people with PAD.

**Recent Findings:**

The ankle-brachial index (ABI) is a non-invasive, guideline-recommended test to diagnose PAD. Circulating biomarkers may offer additional insight into early detection of PAD and risk stratification of adverse events in people with PAD. Observational studies show that higher levels of inflammatory biomarkers (such as C-reactive protein, interleukin-6, soluble intracellular adhesion molecule-1), thrombosis biomarkers (such as D-dimer, fibrinogen), cardiac biomarkers (such as troponin, N-terminal pro-brain natriuretic peptide, lipoprotein A), and omic markers are associated with increased risk of developing PAD. Some of these markers are also associated with severity and adverse outcomes in people with PAD. While association studies have shown links between circulating biomarkers and PAD, no single biomarker or combination of markers is currently guideline-recommended for routine clinical use in diagnosis or management of PAD due to absence of clinical trial data showing that circulating biomarkers definitively improve outcomes in PAD.

**Summary:**

The current state of biomarkers in PAD suggests that further study is needed to determine whether biomarker-guided therapy will improve diagnosis or clinical outcomes for people at risk and people with PAD.

## Introduction

Lower extremity peripheral artery disease (PAD) affects approximately 8.5 million people in the United States, causing significant walking impairment, functional decline, and limb loss [1, 2]. Current diagnostic approaches for PAD rely heavily on clinical presentation and ankle-brachial index (ABI) testing. However, these tools leave gaps in early detection of PAD since 60–90% of people with PAD present with either no exertional leg symptoms or atypical symptoms rather than classic intermittent claudication, making clinical assessment alone challenging for diagnosing PAD [2, 3]. Even after PAD is diagnosed, people with similar clinical presentations or ABIs can have different outcomes with respect to major adverse limb events (MALE) or mortality [1].

People with PAD have higher levels of circulating inflammatory biomle without PAD [4, 5]. Systematic reviews have identified circulating biomarkers such as C-reactive protein (CRP), interleukin-6 (IL-6), fibrinogen, D-dimer, N-terminal pro-brain natriuretic peptide (NT-proBNP) as associated with MALE and mortality in people with PAD [6]. The purpose of this review is to consider whether inflammatory, thrombotic, cardiac, and other types of biomarkers, including proteomic markers, can identify people at elevated risk for PAD or aid in management of adverse outcomes, such as functional decline and MALE, in people with PAD.

## Pathophysiology of PAD

**Fig. 1:**
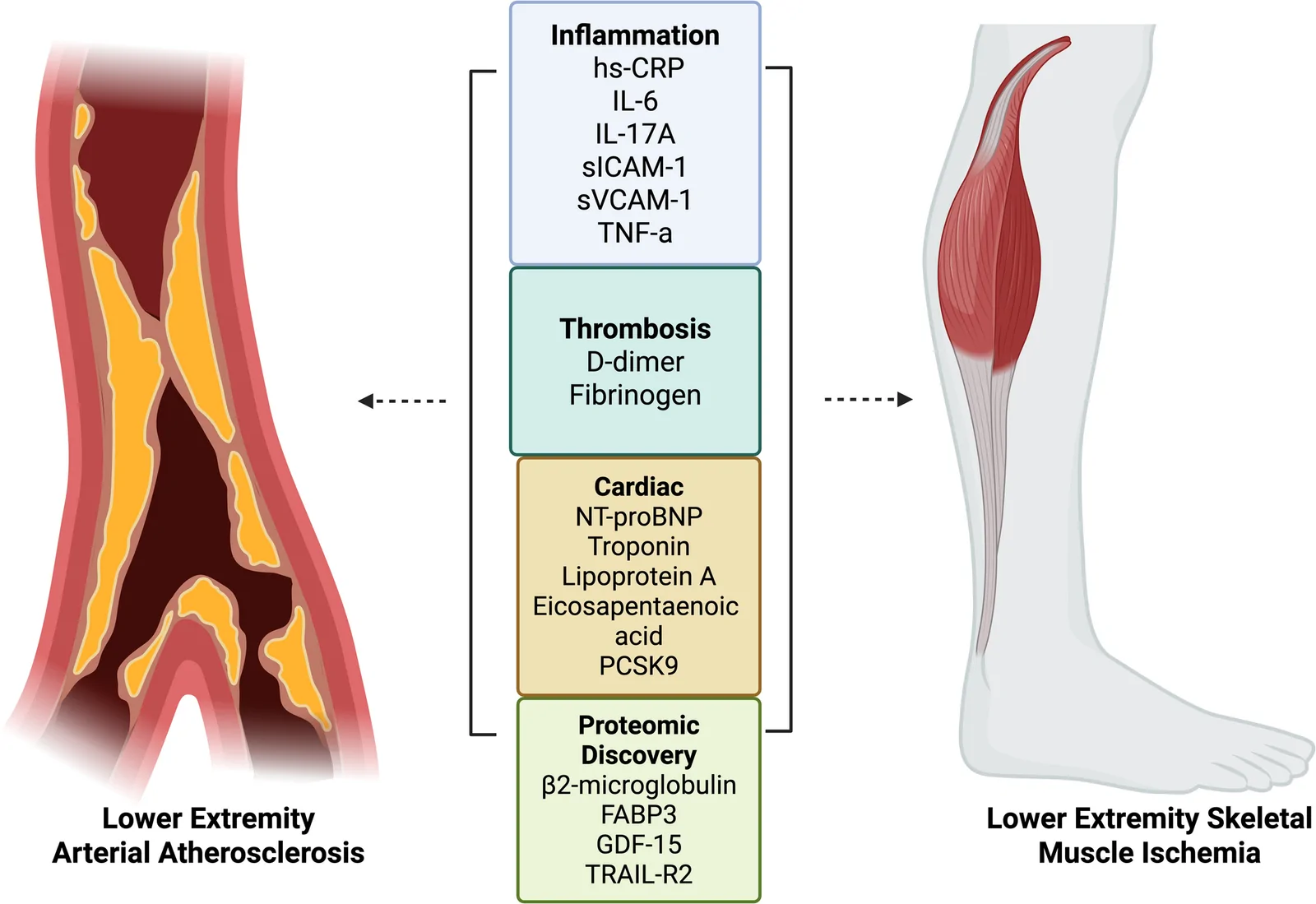
Potential role of circulating biomarkers in lower extremity atherosclerosis and skeletal muscle ischemia in PAD. hs-CRP: High-sensitivity C-reactive protein, IL-6: Interleukin-6, IL-17 A; Interleukin-17 A, sICAM-1: Soluble intracellular adhesion molecule-1, sVCAM-1: Soluble vascular cell adhesion molecule, TNF-a: Tumor Necrosis Factor Alpha, NT-proBNP: N-terminal pro–B-type natriuretic peptide, PCSK9: Proprotein Convertase Subtilisin/Kexin Type 9, FABP3: Fatty Acid-Binding Protein 3, GDF-15: Growth Differentiation Factor 15, TRAIL-R2: tumor necrosis factor-related apoptosis-inducing ligand receptor 2. Figure reference: Thangada, N. (n.d.). Figure 1 - Biomarkers in PAD. Created in BioRender. Thangada, N. (2026) https://BioRender.com/prqyfdm

Understanding PAD pathophysiology is essential for identifying biomarkers that reflect the molecular and cellular mechanisms underlying the disease (Fig. 1) [6]. PAD arises from atherosclerotic disease of the lower extremity due to a combination of endothelial dysfunction, chronic inflammation, oxidative stress, and thrombosis which affects both the lower extremity arterial wall and surrounding skeletal muscle [7, 8]. Atherosclerosis development occurs after endothelial dysfunction leads to atherogenic lipoproteins entering the subintimal space and undergoing oxidation which then triggers an inflammatory response prompting smooth muscle cell migration, neovascularization, and formation of plaque [7, 8]. Over time, macrophages and foam cells release proteolytic enzymes that lead to plaque fissure, instability, and rupture. Ruptured plaque releases contents into the bloodstream which then leads to platelet aggregation and thrombus formation [7, 8]. Unlike in coronary artery disease, acute thrombotic events in peripheral artery disease occur more infrequently since the lower extremity is supplied by many large collateral vessels. However, lower extremity thrombus formation may promote subclinical progression of PAD [6].

## Inflammatory Biomarkers

### Inflammatory Biomarkers and Risk of Developing PAD

Elevated inflammatory biomarkers are associated with an increased risk of developing PAD, with observational studies showing that CRP, IL-6, and soluble intracellular adhesion molecule 1 (ICAM-1) are particularly relevant **(**Table 1**)** [9–11]. Findings from a longitudinal cohort study, the Atherosclerosis Risk in Communities (ARIC), including 9,851 participants free of PAD at baseline who were followed for 17.4 years, showed that high-sensitivity CRP (hs-CRP) was significantly associated with incident PAD (adjusted HR of 1.34 [95% CI 1.18, 1.52] per 1 SD higher hs-CRP) after adjusting for cardiovascular risk factors and another protein, galectin-3 [9]. Galectin-3 was also significantly associated with incident PAD (adjusted HR of 1.14 [95% CI 1.02, 1.27]) per 1 SD higher galectin-3) after adjusting for cardiovascular risk factors and hs-CRP [9]. Findings from another longitudinal cohort study, the Edinburg Artery Study, including 1,519 participants initially free of PAD who were followed for 17 years, found that CRP (adjusted HR of 1.43 [95% CI 1.20, 1.72]), IL-6 (adjusted HR of 1.21 [95% CI 1.06, 1.39]), and soluble ICAM-1 (adjusted HR of 1.20 [95% CI 1.09, 1.32]) were significantly associated with incident PAD when adjusting for age and sex [10]. However, only the association between CRP and incident PAD (adjusted HR of 1.30 [95% CI: 1.08, 1.56]) remained significant after adjusting for cardiovascular risk factors and history of myocardial infarction, stroke, or angina. There was also a statistically significant trend of higher levels of soluble ICAM-1 and worsening PAD severity, defined clinically as presence of intermittent claudication only compared to critical limb ischemia [10].

**Table 1:** Inflammatory & thrombotic biomarkers in peripheral artery disease

Biomarker	Primary Biological Function	Association with Prevalent/Incident PAD	Association with Functional Decline, MALE in PAD
D-Dimer	Thrombosis and inflammation	Elevated D-dimer associated with PAD prevalence [22].	Association between elevated D-dimer and walking impairment was attenuated with adjustment of cardiovascular risk factors; elevated D-dimer associated with thrombotic events in PAD [16, 23–25].
Fibrinogen	Coagulation factor and acute-phase reactant	Elevated fibrinogen associated with PAD prevalence and incidence [10, 22, 24]	Elevated fibrinogen associated with walking impairment; data on fibrinogen in MALE are limited [24].
High-sensitivity C-reactive protein (hs-CRP) and C-reactive protein (CRP)	Acute-phase reactant Systemic inflammation	Elevated hs-CRP and CRP associated with PAD prevalence, incidence, lower ABI, and greater disease severity [9–13].	Elevated hs-CRP and CRP associated with increased MALE, post-revascularization, and mortality [12, 13, 21, 42]
Interleukin 6 (IL-6)	Pro-inflammatory cytokine Upstream regulation of CRP	Elevated IL-6 associated with PAD prevalence and ABI decline [12, 27].	Elevated IL-6 associated with walking impairment, critical limb ischemia, and higher risk of major amputation or death in severe PAD [12, 16, 18, 20, 21].
Soluble intracellular adhesion molecule 1 (sICAM-1)	Endothelial activation and leukocyte adhesion	Elevated sICAM-1 associated with PAD prevalence, lower ABI, and greater disease severity, particularly among Black compared to White individuals [10, 12, 14].	Elevated sICAM-1 associated with walking impairment in PAD; data on MALE are limited [12, 20].
Soluble vascular cell adhesion molecule 1 (sVCAM-1)	Endothelial activation and leukocyte adhesion	Elevated sVCAM-1 associated with PAD prevalence and lower ABI, particularly among Black compared to White individuals [14]. Elevated sVCAM-1 causally associated with PAD in a Mendelian Randomization study [41].	Data on elevated sVCAM-1 and walking impairment in PAD are mixed, with some studies showing an association; data on MALE are limited [16, 18].
Tumor Necrosis Factor Alpha (TNF-α)	Pro-inflammatory cytokine and immune activation	Elevated TNF-α associated with PAD prevalence and greater disease severity [12].	Elevated TNF-α associated with greater walking impairment and MALE following lower extremity revascularization [12, 21].

*Abbreviations*. *PAD *peripheral artery disease, *MALE* major adverse limb event, *ABI *ankle-brachial index

Collectively, these findings suggest that chronic systemic inflammation is associated with PAD development. However, comorbid conditions such as type 2 diabetes mellitus, obesity, cigarette smoking are often seen in people at risk for PAD. Many of the biomarker studies were unable to adjust for these variables, and it is challenging to disentangle the confounding effects of these conditions versus a causal association.

### Inflammatory Biomarkers in People with PAD

Inflammatory biomarkers are elevated in people with PAD, with higher levels correlating with greater PAD disease severity. In a secondary analysis of a randomized clinical trial studying the effects of statins and thiazolidinediones on intermittent claudication, compared to people without PAD, those with PAD had significantly higher levels of circulating TNF-alpha, CRP, soluble ICAM, and IL-6 at baseline [12]. Cross-sectional findings from the National Health and Nutrition Examination Survey (NHANES) (1999–2002), including 4,787 individuals who underwent ABI testing (ABI < 0.9 defined as PAD), showed that compared to those from the lowest quartile of CRP (< 0.05 mg/dL), those from the highest quartile of CRP (> 0. 33 mg/dL) had a greater odds of PAD (adjusted OR 2.14 [95% CI: 1.41, 3.25]) after adjusting for cardiovascular risk factors and physical activity [11]. In an observational study including 387 people with PAD, higher hs-CRP was significantly associated with lower ABI (tertile 1 of hs-CRP < 1.72 mg/L, ABI = 0.70, tertile 2 of hsCRP > 1.72 to < 3.56 mg/L, ABI = 0.65, tertile 3 of hs-CRP > 3.56 mg/L, ABI = 0.57, *P* < 0.01) after adjusting for cardiovascular risk factors and critical limb ischemia [13]. In another observational study including 1,102 Black and 1,013 White people with hypertension (PAD prevalence was 10% among Black and 3.4% among White), Black individuals in the highest quartile of soluble ICAM-1 (> 320 ug/L) and soluble vascular cell adhesion protein (VCAM-1) (> 699 ug/L) had greater odds of PAD compared to those in the lowest quartile [ICAM-1 OR: 5.2 (95% CI: 1.8, 15.2); VCAM-1 OR: 2.2 (95% CI: 1.0, 4.8)] after adjusting for cardiovascular risk factors [14]. The association between soluble VCAM-1 and odds of PAD was slightly attenuated with the addition of CRP and fibrinogen to cardiovascular risk factors (adjusted OR: 2.0 [95% CI: 0.9, 4.4]) [14]. The association between soluble ICAM-1 and VCAM-1 was not observed among White individuals [14]. The observed race-ethnic difference in soluble ICAM-1 and VCAM-1 and PAD risk is not well-understood, but may be a result of single nucleotide polymorphism (SNP) in ICAM-1 (rs5491 A/T) gene [15]. Table 1 summarizes the inflammatory biomarkers commonly elevated in people with PAD.

Overall, these findings suggest that inflammatory biomarkers are elevated in people with PAD and associated with greater disease severity. While inflammatory biomarkers may inform risk stratification for people with PAD in the future, additional work is needed to understand population-level differences in inflammatory biomarkers among various race-ethnic groups.

### Inflammatory Biomarkers and Walking Impairment in PAD

Elevated inflammatory biomarkers are associated with walking impairment and functional decline in people with PAD **(**Table 1**)**[16–18]. Findings from a cross-sectional study including 423 people with PAD showed that compared to people with the lowest quartile of D-dimer, IL-6, soluble VCAM-1, CRP, homocysteine, and soluble ICAM-1, those from the highest quartile had a significantly lower 6-minute walk distance after adjusting for ABI, cardiovascular risk factors, osteoarthritis, spinal stenosis, cancer, and pulmonary disease [16]. The associations between CRP, soluble ICAM-1, and 6-minute walk were no longer significant after adjusting for calf-muscle area, a potential confounder in the relationship between the biomarkers and functional impairment [16, 17]. In a cross-sectional study including 75 people with PAD, higher levels of TNF-alpha, CRP, IL-6, and soluble ICAM-1 were significantly associated with worse maximum walking time compared to those with lower levels of these inflammatory biomarkers after adjusting for age, gender, smoking, and ABI [12]. Additionally, higher TNF-alpha gene expression in peripheral blood monocytes was also associated with worse maximum walking time [12]. Findings from a prospective cohort study including 255 people with PAD found that those from the highest tertile of IL-6 levels had significantly greater decline in 6-minute walk distance compared to those from the lowest tertile at 1-year follow-up (−78.6 feet vs. −21.4 feet) after adjusting for ABI, cardiovascular risk factors, and physical activity [18]. Similarly, those in the highest tertile of IL-6 had significantly greater decline in Short Physical Performance Battery scores compared to those in the lowest tertile at 1-year follow-up (−0.62 vs. −0.18) after adjusting for ABI, cardiovascular risk factors, and physical activity [18].

The mechanism by which elevated circulatory inflammatory biomarkers lead to greater walking impairment in PAD likely involves both progression of lower extremity atherosclerotic disease and direct inflammatory effects on skeletal muscle (Fig. 1) [17]. For instance, in a longitudinal cohort study including 423 people with PAD, higher levels of D-dimer, CRP, IL-6, and soluble VCAM-1 were significantly associated with smaller calf muscle area after adjusting for cardiovascular risk factors and physical activity [17]. Higher levels of soluble VCAM-1 were also associated with lower calf strength after adjusting for cardiovascular risk factors [17].

Together, these findings support an association between inflammatory biomarkers and functional decline in PAD. However, existing studies do not establish the utility of longitudinal tracking of inflammatory biomarkers to monitor functional decline in PAD. Additional studies are needed to determine if these biomarkers can serve as clinical monitoring tools for people with PAD at high risk for walking impairment.

### Inflammatory Biomarkers in Critical Limb Ischemia (CLI) and MALE

Elevated inflammatory biomarkers are associated with CLI and MALE in people with PAD **(**Table 1**)**. In a prospective cohort study including 119 people with PAD (65 with intermittent claudication and 54 with CLI), levels of 27 inflammatory cytokines were measured at the time of enrollment. Those with CLI had significantly higher levels of multiple inflammatory cytokines (IL1- receptor agonist, IL-6, IL-8, IL-12, p70, G-CSF, IP-10, MCP-1, MIP-1alpha, PDGF-beta, RANTES, and TNF-alpha) compared to those with intermittent claudication alone [19]. Additionally, those with CLI who required a major amputation had significantly higher basic fibroblast growth factor (FGF-basic) compared to those with CLI who did not require major amputation (median FGF-basic: 49.04 pg/mL vs. median FGF-basic: 33.04 pg/mL) [19]. In a secondary analysis of a randomized clinical trial including people with severe PAD (ABI < 0.6) and healthy subjects without PAD, every 1-unit (log-scaled) increase in IL-6 (HR: 1.35 [95% CI 1.06–1.71]) and IP-10 (HR: 1.49 [95% CI 1.13–2.00]) was significantly associated with greater hazard for major amputation or death after adjusting for age, sex, glomerular filtration rate, diabetes, hemoglobin, ABI, Fontaine classification, and CRP levels [20]. In an observational study of 299 people with PAD who underwent lower extremity angioplasty, those with highest levels of IL-6, CRP, and TNF-alpha at the time of enrollment had higher rates of post-angioplasty MALE at 12-months compared to those with the lowest levels of these biomarkers after adjusting for cardiovascular risk factors and Rutherford staging of chronic limb ischemia [21].

Overall, inflammatory biomarkers are associated with MALE, suggesting that inflammation may play a role in the transition from stable PAD to limb-threatening ischemia. Additional studies are needed to determine whether these inflammatory biomarkers may serve a prognostic role in identifying people with PAD at high risk for CLI.

## Thrombosis Biomarkers

D-dimer and fibrinogen concentrations in blood are elevated in people with PAD, with higher levels associated with more severe disease, walking impairment, and future thrombotic events (Table 1) [5, 22, 23]. Findings from a cross-sectional analysis from the ARIC study, which included 441 people with PAD and 13,939 people without PAD, showed that compared to people in the lowest quartile of fibrinogen, those in the highest quartile had a greater odds of PAD (OR for men: 3.49 [95% CI: 1.68, 7.26 ], OR for women: 2.44 [95% CI: 1.58, 3.77]) after adjusting for cardiovascular risk factors [22]. Additionally, compared to people from the lowest quartile of D-dimer, those from the highest quartile of D-dimer had a greater odds of PAD (OR: 2.70 [95% CI 1.56, 4.65]) after adjusting for sex and cardiovascular risk factors [22]. Prospective findings from the Edinburg Artery Study, including 1,519 participants initially free of PAD who were followed for 17 years, found fibrinogen was significantly associated with incident PAD (OR: 1.16 [95% CI 1.05, 1.17]), but D-dimer was not [10]. In a cross-sectional study of 127 people with PAD, elevated D-dimer (*r*= −0.31), tPA antigen (*r*= −0.19), and fibrinogen (*r*= −0.20) were all significantly inversely associated with maximum treadmill walking distance [24]. However, after adjustment for cardiovascular risk factors and homocysteine, only fibrinogen remained significantly inversely associated with maximum treadmill walking distance and ABI [24]. In a prospective study including 123 people with PAD followed 4.2 years for incident thrombotic events, 38 events occurred [23]. Relative risk of thrombotic events (defined as sudden cardiac death, myocardial infarction, ischemic stroke, acute thrombosis in any vascular bed diagnosed by angiography or vascular ultrasound) was significantly higher among those from the highest quintile of D-dimer (> 861 ng/mL) compared to the lowest quintile after adjusting for age and other cardiovascular risk factors (RR: 14.1 [95% CI 1.7, 115.9]) [23]. In another prospective study including 595 people with PAD followed for 3 years, mean D-dimer was significantly higher 2-months prior to an ischemic heart disease event (defined as myocardial infarction, unstable angina, or death related to ischemic heart disease) compared to levels obtained at timepoints 10–32 months prior to the event [25].

The mechanisms underlying these associations may reflect both prothrombotic and inflammatory processes. For instance, fibrinogen facilitates atherosclerosis development through multiple pathways such as increased endothelial permeability, monocyte recruitment, smooth muscle migration, and enhanced platelet reactivity [26]. However, both fibrinogen and D-dimer are also acute phase reactants and inflammatory markers. Thus, the underlying pathogenesis of PAD development and progression may also be linked to the inflammatory effects of these biomarkers [27].

## Cardiac Biomarkers

Troponin, a protein found in cardiac and skeletal muscle, NT-proBNP, a protein released due to heart muscle strain and stretch, and Lipoprotein A [Lp(a)], an atherogenic cholesterol molecule, are common cardiac biomarkers that are elevated in people with PAD and may also serve as markers for PAD severity, MALE, and mortality (Table 2) [28–32].

**Table 2:** Cardiac biomarkers in peripheral artery disease

Biomarker	PrimaryBiological Function	Association With PAD	Association With Adverse Outcomes in PAD
High-sensitivity cardiac troponin (hs-cTnT / hs-cTnI)	Marker of subclinical myocardial injury	Elevated hs-troponin associated with PAD prevalence, incidence, and worse ABI, even in the absence of coronary artery disease [28–30].	Elevated hs-troponin associated with increased risk of all-cause mortality and MALE in PAD [28, 29, 31].
NT-proBNP: N-terminal pro-brain natriuretic peptide	Marker of cardiac wall stress and neurohormonal activation	Elevated NT-proBNP associated with PAD prevalence and incidence [28, 30, 31].	Elevated NT-proBNP associated with all-cause mortality and MALE in PAD [28].
Lipoprotein(a) [Lp(a)]	Pro-atherogenic and pro-inflammatory lipoprotein	Elevated Lp(a) levels associated with PAD incidence [32–34]. Elevated Lp(a) causally associated with PAD in a Mendelian Randomization study [41].	Elevated Lp(a) associated with increased risk of MALE [34].
Eicosapentaenoic Acid (EPA)	Omega-3 fatty acid with anti-inflammatory, anti-atherogenic properties	Elevated EPA levels associated with lower risk of PAD [43]. Elevated EPA causally associated with PAD in a Mendelian Randomization study [41].	Data on circulating EPA and adverse outcomes in PAD is limited. In a randomized clinical trial of EPA supplementation in PAD, compared to control, those receiving EPA had fewer major adverse coronary events [44].
Proprotein Convertase Subtilisin/Kexin Type 9 (PCSK9)	A pro-atherogenic protein involved in LDL receptor recycling	Elevated PCSK9 levels associated with higher risk of PAD even after adjusting for Lp(a) [45]. Elevated PCSK9 causally associated with PAD in Mendelian Randomization study [41].	Data on circulating PCSK9 and adverse outcomes in PAD is limited. In a randomized clinical trial of PCSK9 inhibition in people with and without PAD, compared to the control, those receiving Evolocumab had significantly less MALE in both people with and without PAD [46].

*Abbreviations. PAD *peripheral artery disease, *MACE* major adverse cardiac event, *MALE *major adverse limb event

### Troponin and NT-proBNP

In a meta-analysis of 8 observational cohort studies including 5,313 people with PAD followed for a median of 27 months, troponin was elevated (> 99th percentile value) in 5.3% of people with intermittent claudication and 62.6% of people with CLI [29]. In people with PAD, elevated troponin was also associated with greater all-cause mortality (pooled HR: 2.85 [95% CI: 2.28, 3.57]) [29]. In a cross-sectional study of NHANES participants from 1999 to 2004 (*N* = 6,098), 348 people had PAD (prevalence 4.1%), defined as right or left-sided ABI < 0.90 [30]. Of those with PAD and no other cardiovascular disease, the prevalence of elevated NT-proBNP (≥ 125 ng/L), hs-troponin T (≥ 6 ng/L), and hs-troponin I (≥ 6 ng/L for men, ≥ 4 ng/L for women) was 54.0 ± 3.4%, 73.9 ± 3.5%, and 32.3 ± 3.7%, respectively [30]. Additionally, compared to people without PAD with the lowest levels of NT-proBNP (< 125 ng/L), those with the highest levels of NT-proBNP (≥ 450 ng/L) had a greater odds of PAD (OR: 2.68 [95% CI: 1.73, 4.16]), after adjusting for cardiovascular risk factors and chronic kidney disease [30]. However, hs-troponin T and hs-troponin I were not significantly associated with greater odds for PAD [30].

Prospective findings from the ARIC study, which included 12,288 middle-aged adults followed for 22 years, 454 people developed PAD with 164 cases of CLI [28]. People from the highest category of high-sensitivity cardiac troponin T (hs-cTnT) (≥14 ng/L vs. < 3 ng/L) and NT-proBNP (≥258.8 pg/mL vs. < 51.5 pg/mL) had greater hazards for PAD (HR for hs-cTnT: 2.84 [95% CI: 2.02, 4.00], HR for NT-proBNP: 3.16 [95% CI: 2.23, 4.49]) and for CLI (HR for hs-cTnT: 7.74 [95%. CI: 4.43, 13.55], HR for NT-proBNP: 4.63 [95% CI: 2.61, 8.23]). These associations persisted after adjustment for cardiovascular risk factors, ABI, and NT-proBNP in the troponin model and hs-cTnT in the NT-proBNP model [28].

In a prospective cohort study of 95 people with PAD (77% male) who were followed for 10 years, hs-TnT and NT-proBNP were measured at baseline to determine the prognostic performance of these measures on life expectancy [31]. In multivariable Cox regression models, the association between hs-TnT and mortality was statistically significant (HR: 1.93 [95% CI: 1.33, 2.79] after adjusting for age, gender, prior cerebral artery disease, and diabetes [31].

### Lipoprotein(a)

Findings from the Copenhagen General Population Study, which included 108,146 people, evaluated the association between Lp(a) and incident PAD [33]. A total of 2,450 people developed PAD in the cohort over a median of 7.4 years follow-up. Elevated Lp(a) levels > 99th percentile (≥143 mg/dL, ≥307 nmol/L) vs. < 50th percentile (≤ 9 mg/dL, ≤ 17 nmol/L) were associated with greater risk of PAD (32/681 events vs. 272/17,424 events; HR 2.99, [95% CI: 2.09, 4.30]) after adjusting for cardiovascular risk factors and menopause status and use of hormone replacement therapy among women [33]. In findings from a UK Biobank prospective cohort study including 460,544 people with available Lp(a) measurements (of whom 3,701 had prevalent PAD), 6,347 (1.4%) developed incident PAD over a median follow-up of 13.6 years [34]. People with prevalent PAD who had an elevated Lp(a) (> 150 nmol/L) had a 1.57 times greater risk of developing a MALE than people with PAD with a normal Lp(a) after adjusting for age, sex, and ancestry [34]. Additionally, median Lp(a) concentrations were significantly higher in people with PAD (25.3 nmol/L vs. 19.5 nmol/L) and people with PAD complicated by MALE (33.2 nmol/L vs. 19.5 nmol/L) compared to those without any atherosclerotic vascular disease [34].

While cardiac biomarkers such as troponin, NT-proBNP, and Lp(a) are associated with PAD, it is unclear whether routinely testing for these biomarkers will change clinical outcomes in people with PAD. Additional studies are needed to determine whether these cardiac biomarkers can risk stratify people at high risk for incident PAD while providing prognostic information about MALE and mortality in those with prevalent PAD [1].

## Biomarkers Identified Through Proteomic Discovery

The circulating proteome consists of the proteins present in the plasma and serum of an individual. Multiple circulating proteins have been associated with PAD severity and adverse outcomes (Fig. 1)[35]. A proteomic study from 2007 including 45 people with PAD and 43 people without PAD showed that of the 1,619 proteins included in the analysis, plasma beta2-microglobulin was significantly elevated in people with PAD compared to people without PAD (2.17 ± 0.63 µg/mL vs. 1.72 ± 0.42 µg/mL) independent of risk factors including coronary artery disease [36]. Additionally, plasma beta2-microglobulin levels were inversely correlated with ABI [36]. In a prospective cohort study including 374 people with PAD and 184 people without PAD, levels of plasma fatty acid binding protein 3 (FABP3) were significantly higher in people with PAD compared to without PAD (median = 3.27; IQR 2.35–4.46 vs. median = 1.74; IQR 1.25–2.55) [37]. Additionally, Cox regression analysis showed that FABP3 > 1.55 ng/mL may independently predict MALE (need for arterial revascularization or major limb amputation) and decline in ABI > 0.15 in people with PAD over 3-year follow-up [37]. The exact role of FABP3 in PAD is unknown and additional studies are needed to determine its clinical significance.

The advent of high-throughput proteomic analyses has expanded our ability to identify multiplex proteomics relevant to PAD. In a study including 565 people with carotid and/or lower extremity PAD from two separate cohorts (*N* = 436, discovery cohort; *N* = 129, validation cohort), the association between 81 serum proteins and all-cause mortality was evaluated [38]. The results showed that growth differentiation factor 15 (GDF-15) (HR: 1.57, [95% CI: 1.18–2.08]) and tumor necrosis factor-related apoptosis-inducing ligand receptor 2 (TRAIL-R2) (HR: 1.40 [95% CI: 1.06–1.84]) were significantly associated with increased all-cause mortality in the discovery cohort during 10-year follow-up. These models were adjusted for age, ABI, estimated glomerular filtration rate, and cardiovascular risk factors. Adding GDF-15 and TRAIL-R2 to the COPART score, a validated clinical tool that can predict 1-year cardiovascular death in PAD, significantly improved all-cause mortality discrimination compared to COPART score alone in the discovery cohort [38]. While these proteomic associations are promising, additional studies are needed to confirm whether these proteomic biomarkers can be utilized for either predicting risk of incident PAD or risk of MALE and mortality among people with PAD.

## Future Directions

### Multimarker Approaches

Multimarker approaches integrating biomarkers across multiple pathways such as inflammation, thrombosis, oxidative stress, may better capture the multifactorial pathophysiology of PAD within a single risk assessment. In a prospective cohort study of 569 people with PAD followed for 3 years for primary outcome of MALE, three machine learning models (Extreme Gradient Boost [XGBoost], random forest, and decision trees) were trained to use demographic, clinical, and biomarker data at baseline to predict MALE at 3 years [39]. Biomarkers included plasma NT-proBNP, FABP3, and fatty acid binding protein 4 (FABP4), which were measured at baseline. A total of 162/569 (26%) of people developed MALE by 3-years [39]. The XGBoost machine learning model best predicted 3-year MALE (AUROC = 0.88 [95% CI: 0.84, 0.94], sensitivity 88%, and positive predictive value of 83%) [39]. In an independent validation cohort, XGBoost reached an AUROC of 0.87 [95% CI: 0.82, 0.90] [39]. The 10-most important variables associated with 3-year MALE were (in order of decreasing significance): FABP3, FABP4, age, NT-proBNP, active smoking, diabetes, hypertension, hyperlipidemia, coronary artery disease, and sex [39].

These findings highlight the potential for integrating a multi-marker approach to common clinical variables known to be significant in PAD to allow for prediction of adverse outcomes like MALE. Barriers to implementation in clinical practice include challenges with standardizing biomarker thresholds, validating panels across diverse populations, and demonstrating cost-effectiveness.

### Biomarkers and PAD: Can we Establish a Causal Link?

Multiple circulating biomarkers spanning diverse biological pathways are associated with incident PAD, prevalent PAD, PAD severity, and MALE. However, whether these biomarkers are causally associated with PAD remains unclear. For instance, one inflammatory biomarker, galectin-3, has been associated with incident PAD in an observational study [9]. However, a 2-sample Mendelian randomization study including 212,453 people of East Asian ancestry (3,593 with PAD, 208,860 without PAD) did not identify a causal relationship between galectin-3 and PAD [40]. Another 2-sample Mendelian randomization study including 243,060 people (31,307 with PAD) from European, African, and Hispanic ancestry evaluated the association between 204 plasma biomarkers with known links to PAD and the effect of genetically predicted variation in each biomarker on PAD [41]. Of the 204 biomarkers, only 10 were significantly associated with PAD. The findings showed that biomarkers in the lipid regulation pathway, such as Lp(a), eicosapentaenoic acid (EPA), PCSK9, and biomarkers in the inflammation pathway, such as beta2-microglobulin, IL-17 A and soluble VCAM-1 were potentially causally associated with PAD [41]. Notably, inflammatory biomarkers such as hs-CRP, IL-6 and cardiac biomarkers such has hs-cTnT, NT-proBNP were not significantly associated with PAD in Mendelian Randomization.

In a randomized clinical trial including 38 people with PAD randomized to receive canakinumab, an IL-1β inhibitor which reduces inflammation associated with atherosclerosis, versus placebo for 12 months, hs-CRP and IL-6 significantly decreased after 1 month of treatment in those receiving canakinumab compared to placebo. However, there was no significant difference in the primary outcome of superficial femoral artery plaque burden between the canakinumab and placebo group at 12-months. Those receiving canakinumab compared to placebo had significantly greater improvement in an exploratory outcome of 6-minute walk distance up to 9 months (between group difference: 50 m [80% CI: 2.6, 97.0]), but not thereafter at 12 months. These findings show that anti-inflammatory therapies may lower systemic inflammation but the exact effect on PAD progression and outcomes is unclear. Additional studies are needed to confirm whether biomarkers that are commonly elevated in PAD are causally associated with PAD.

## Conclusions

Multiple observational studies show associations between circulating inflammatory, thrombosis, cardiac biomarkers and incident PAD as well as severity of disease and risk of MALE in people with PAD. Despite this evidence, the 2024 American College of Cardiology/American Heart Association clinical practice guidelines for lower extremity PAD do not recommend measurement of circulating biomarkers for PAD screening, diagnosis, or risk stratification of MALE [1]. This may be due to lack of randomized clinical trials demonstrating that biomarker-guided screening and management definitively improves clinical outcomes in PAD. Potential next steps for biomarkers in PAD include validation and standardization of biomarker thresholds across various sex and race-ethnic groups followed by randomized trials determining whether biomarker-guided therapy can improve clinical outcomes in PAD.

## Key References

- Gornik HL, Aronow HD, Goodney PP, Arya S, Brewster LP, Byrd L, et al. 2024 ACC/AHA/AACVPR/APMA/ABC/SCAI/SVM/SVN/SVS/SIR/VESS Guideline for the Management of Lower Extremity Peripheral Artery Disease: A Report of the American College of Cardiology/American Heart Association Joint Committee on Clinical Practice Guidelines. Circulation. 2024;149(24):e1313-e410.
  - ○  The 2024 PAD guideline statement supported by multiple professional societies recommends diagnosing PAD by clinical history and ankle-brachial index. There is no role for routinely measuring circulating biomarkers either for diagnosis or management of PAD.
- Sharma P, Klarin D, Voight BF, Tsao PS, Levin MG, Damrauer SM. Evaluation of Plasma Biomarkers for Causal Association With Peripheral Artery Disease. Arterioscler Thromb Vasc Biol. 2024;44(5):1114-23.
  - ○  This study evaluated whether circulating biomarkers previously identififed in observational studies as associated with PAD were causally-associated with PAD using two-sample Mendelian randomization. Their findings showed that many of the inflammatory and thrombotic biomarkers shown to be associated with PAD were not causally-associated with PAD and instead biomarkers involved in lipid regulation (ie: Lipoprotein A, Eicosapentaenoic Acid) were causally associated with PAD.

## Data Availability

No datasets were generated or analysed during the current study.
